# The care of older people with depression in Nigeria: qualitative exploration of the experience of lay providers in primary care settings

**DOI:** 10.1002/gps.6147

**Published:** 2024-09-01

**Authors:** Akin Ojagbemi, Stephanie Daley, Yvonne Feeney, Oye Gureje

**Affiliations:** 1 World Health Organization collaborating centre for research and training in mental health, neuroscience, and substance abuse, Department of psychiatry, College of Medicine, University of Ibadan, Nigeria; 2 Centre for Dementia Studies, Brighton and Sussex Medical School, Brighton, UK

**Keywords:** Comorbidities, Ethnogerontology, Health disparities, Home healthcare, Late-life depression, Low- and middle-income countries, Psychosocial interventions, Task sharing, Stigma, Underserved populations

## Abstract

**Objectives:**

There is a large treatment gap for mental health conditions in sub-Saharan Africa where most patients who receive any care do so from lay primary health care workers (PHCW). We sought to examine the experiences of PHCW who provide care for older people with depression in Nigerian primary health care (PHC) settings.

**Methods:**

Qualitative study design. A total of 24 PHCW participated. Using in-depth key informant interviews (KIIs), we explored the views of 15 PHCW selected from 10 rural and urban PHCs in South-Western Nigeria. An additional focus group discussion comprising nine participants was also conducted to discuss emerging themes from KIIs. Data were analysed using thematic analysis.

**Results:**

Three overall themes were identified: views about depression, treatment options, and community outreach implications. Participants perceived depression in older people as being characterized by a range of mood, behavioural, and cognitive symptoms which made clinical assessments particularly challenging. Common treatment options used by PHCW included general advice and counselling, as well as frequent need to prescribe mild analgesics, vitamins and occasional sedatives in line with patients’ expectations. Antidepressants were rarely used even though PHCW are authorized. While home visits are part of their expected work schedule, PHCW rarely implemented these due to non-availability of transport facilities. Mobile technology was identified as a possible way of overcoming this constraint to providing community based mental healthcare for older people.

**Conclusion:**

PHCWs perceived that patients’ poor cognitive performance, expectations to prescribe sedatives, analgesics and vitamins, as well as non-existence of community-based services were existing barriers to providing evidenced based continued care for older people with depression in the study settings.

## Introduction

Approximately 212 million Africans will be 60 years or older by 2050(1), and Nigeria is the African country with the largest population of older people(2). Depression is the most common and disabling mental health condition in old age(3). The rate of late-life depression in Nigeria is projected to be among the highest globally(4). Yet, most people living with late-life depression in Nigeria have no access to treatment(5). This is, in part, because the condition is rarely detected in the community(4, 6). Even when detected, mental health referrals for hospital treatments are rarely taken up due to the limited number of mental health specialists, the stigma attached to mental health consultation, and the physical and material limitations of old age(7),(8).

The majority of Nigerians who seek treatment for depression do so from non-physician primary health care workers (PHCW)(9). The main PHCW in Nigeria are community Health Officers (CHO) and Community Health Extension Workers (CHEWs). They are more readily available and often live in the same community as patients(10). These workers receive 2-3 years of post-high school education in the treatment of common conditions presenting in Primary Health Care (PHC). The official clinical guidelines used routinely by PHCW in Nigeria is the ‘Standing Order’. The ‘Standing Order’ is a written protocol informed by the WHO Package of essential non-communicable Disease Intervention for PHC in Low-Resource Settings(11). This classification and treatment guide authorizes PHCW to prescribe a limited range of mental health medications including chlorpromazine antipsychotic and amitriptyline antidepressant. However, PHCW in Nigeria have generally not received training in the assessment and treatment of late life depression. Yet, PHCW have the potential to be a critical resource in bringing evidence-based depression treatment to vulnerable older Nigerians.

To achieve this aspiration, it is essential to understand the views, context and constraints of PHCW who provide care for older people with depression in Nigeria. The present study was conducted as a part of the National Institute of Health and Care Research (NIHR) and Wellcome Trust funded **In**tervention for **D**epression **i**n underserved **G**eriatric p**o**pulations (INDIGO Project). The specific objectives of the current sub-study were to explore: 1). Barriers and facilitators faced by PHCWs when providing care for older people with depression, and 2). Perceptions regarding home-based care for older people with depression and other mental health conditions.

## Methods

### Study design

We chose a qualitative design to allow for a more in-depth exploration of the personal experiences and views about late-life depression beyond what might be possible to elicit in a quantitative survey(12). These, in the present study included facilitators and barriers to care of an older person with depression and perception about home-based care for older people. This information was gathered using Key Informant Interviews (KII) and a Focus Group Discussion (FGD).

### Participants and Settings

The study took place in 10 PHCs purposively selected to represent a range of urban and rural settings within five local government areas in Ibadan metropolis, Southwestern Nigeria. In all, Ibadan metropolis has a population of approximately 3.5 million people. There are 186 PHCs in Ibadan each serving a population of approximately 10,000. Participants were purposively selected CHOs and CHEWs who had a recent experience of caring for an older person with depression in any of the 10 PHCs.

### Study procedure

All participants in the present study provided informed and written consent to participate. We first conducted 15 semi-structured KIIs with CHOs (n=11) and CHEWs (n=4). This was to explore personal experiences. We next conducted a FGD comprising nine informants (3 CHOs and 6 CHEWs). FGD was conducted to further explore interview themes.

### Topic guides

We developed two contextually relevant interview topic guides for both the FGD and KIIs (Supplementary Tables I and II). Interview guides were iteratively reviewed by a group comprising 3 experienced Nigerian research assistants (RAs) and 3 experts in older peoples’ mental health and education. The topic guide was reviewed following interviews with the first two participants. The interviews explored a range of topics such as daily work routines, personal experiences, perception about older peoples’ depression, identifying key problems in assessment and treatment of older people with depression, as well as thoughts about home-based care models for older people with depression. The FGD guide was developed taking account of observations during interviews. FGD explored interview themes, as well as organizational, contextual and process issues.

The FGD and KIIs were conducted between February and June 2022, in Yoruba and English languages, at the PHCs (Table 1), and by trained RA under the supervision of SD and AO. Participants covered the range of groups of interest (14 CHOs and 10 CHEWs); 18 females and six males (Table 2). While KIIs lasted approximately 45 minutes, the FGD lasted for an hour and half. All sessions were audio-recorded using Amplivox audiotape recorder.

### Qualitative data analyses

Recordings of FGD and KIIs were transcribed verbatim by research staff and anonymised to maintain confidentiality. Translation into English was caried out by three independent forward translators fluent in English and Yoruba languages. They compared their versions to identify discrepancies, use of ambiguous or vague wording and accuracy. A final version of the transcript in English was developed with the three original translators by a research staff fluent in English and Yoruba languages. Back translation to the source language was conducted by a fourth independent translator who was blinded to the original transcript.

All final transcripts were analysed in three stages using thematic analysis(13) (Figure I). Analyses started with three researchers (AO, SD and YF) independently reading and re-reading transcripts for familiarity and to begin to identify pertinent themes. Each researcher labelled meaningful segment of text manually using descriptive codes. The first four KII transcripts were independently coded. The researchers met after each transcript was coded to review their respective preliminary codes to identify areas of similarities and differences. Disagreements in assignment of attribute codes was resolved through discussion to achieve consensus. A draft initial coding framework was then developed, reviewed, and agreed upon.

In the second phase, a further 10 transcripts were coded using the initial coding framework. Systematic collation and review of data within each code was enabled by the computer software package, NVivo 10 (QSR International, 2012). The qualitative data set was reviewed during frequent meetings between the researchers during which respective codes within new transcripts were compared with previous transcripts for rigor(14). A focussed coding framework was then developed in the third phase. Relationships between codes were also identified at this stage. The final phase of the analysis included final interviews, from which four key themes were identified for both lived experience participants and caregivers. These were reduced to three overall themes after review by the research team. Relevant adaptation of this approach was used in the analyses of the FGD.

## Results

### Key themes

The analyses of the interviews and FGD generated three main themes relating to depression in older people attending PHC: views about depression, treatment options and community outreach implications (Table 3).

### Theme 1: Views about older people with depression

This theme is related to PHCW experience in assessing older people with depression who present in PHC and include their perception about condition, observations about its symptoms, accessing of care and perceived triggers.

### Symptoms and presentation

Primary healthcare workers stated that many older people presenting with depression reported mood changes as well as bodily pains and other physical health symptoms. The health workers held a perception that these conditions led to the display of challenging behaviours such as undue irritability and impatience during clinical consultation. They viewed these rapid changes in emotional states as well as a history of recent social withdrawal as key symptoms of depression in older people who present in primary care.

*‘In a depressive (depressed) elderly, they usually have low mood, mood swings and withdraw(al) from daily activities. So, the one with depression is withdrawn and experiences low mood…, non-depressed person don’t show these symptoms.’*

**(39 years old CHEW)**

In the view of PHCW, older people often had depression symptoms for weeks without seeking medical help. A lack of awareness of the medical nature of depression symptoms such as mood and behavioural changes was perceived by PHCW as the main reason for non-presentation in primary care. PHCWs reported that patients and their families only sought help in primary care when the older person developed persistent and distressing physical health symptoms as part of depression.

*‘He wasn't violent, but he withdrew and was not eating. He has been on this for more than 2 months. But they didn't know until he came to the clinic presenting a physical problem that it was detected that he had depression.’*

(**56 years old CHO**)

### Precipitating factors

PHCWs reported that many older people with depression were observed to have insufficient social and caregiving support. Health workers remarked that immediate family members and adult children were the most important source of support for older people in Nigeria. However, participants reported that it was increasingly common for these family members to work outside the home and were therefore not often available to provide day to day support. PHCWs suggested that depression in older people was often caused by the resulting unmet expectation of support from immediate family members.

*‘The difference I know is that when you ask them about their children, they (i.e*., *the older person) would tell you that they (live) are alone. That (information) would suggest to us that she has depression in the sense that there is no one to care for her….’*

(**41 years old CHEW**)

PHCWs also reported that personal economic challenges may be another trigger of depression in older people, in particular unemployment and lack of family or governmental financial support. Participants identified a general lack of social welfare programmes and safety nets initiatives for older people as being a contributing factor.

*‘There was a woman who suffered economic reversals in her business. She could no longer care for her children, so this made her depressed.’*

(**48 years old CHO**)

### Perceived difficulty providing care for older people with depression

PHCWs reported challenges in working with older people with depression because of a perception that their behaviour was difficult. PHCWs reported that difficult behaviours were commonly displayed when they attempted to challenge patients’ understanding of their own illness experience including causal interpretations. Participants reported that patients viewed their depression as related to socio-cultural or religious factors, in comparison to the PHCW biomedical review of depression.

*‘I actually realize that most of the elderly, they have issues of their belief system. No matter what you try to tell them, they have a concept of what is wrong with them. They have this mindset of what is wrong with them which they want to tell you and they want u to believe it too. So, you need to find a way of trying to convince them to change their orientation about that thing that they believe. Sometimes they believe that it’s spiritual. Yes, “the awon aye” (A curse or bewitchment)*..*maybe from their families*.

(**38 years old CHO**)

Participants reported that some patients displayed high sensitivity to PHCW questioning or approach to clinical consultation. One area which was strongly highlighted was the challenge of discussing suicidal thoughts or intentions, which could lead to anger or withdrawal form treatment.

*‘Hope you are not thinking of commuting suicide? that is the question they react to most of the time. It is always irritating to them, most of them will be like God forbid.’*

(**37 years old CHEW**)

Memory was also seen as a symptom of depression in older people. Participants suggested that memory loss was commonly associated with depression in older people. PHCWs reported that older people commonly had difficulty in remembering questions during clinical encounters and instead provided unusually detailed responses that were considered by the PHCW as irrelevant.

*‘…she doesn't talk well, if asked a question, she derails completely from the answer.’*

(**38 years old CHO**)

PHCWs reported that it was difficult for them to differentiate between older people with depression from those with dementia because of a perceived overlap in symptoms. In the view of many PHCW, mood, behavioural and cognitive symptoms observed in older people with depression demonstrated substantial similarities with those they observed in people with dementia. In this way, many PHCW noted that there was often a risk of misdiagnosing older patients with depression as having dementia.

*‘When someone gets old, they forget things. This is called senile dementia. But if someone does not have the knowledge of depression, he might mistake or misdiagnose it (Dementia) as depression.’*

**(51 years old CHO)**

### Theme 2: Treatment of depression in PHC

This theme is related to PHCW experience in treating and providing support older people with depression who present in PHC and includes views about available treatment options, facilitators and barriers of effective treatment and support.

### Treatment options

A common treatment option for depression reported by PHCWs was counselling. This was provided in the form of basic psychoeducation and advice. Counselling for depression in older people was reported to span over several follow-up sessions, and until PHCW observed symptomatic improvement. The sessions were reported to be typically discussive and involved the use of self-disclosure and narratives of personal experiences, as well as challenging of patients’ beliefs and misconception about depression. Health workers viewed counselling as an effective treatment for depression but expressed frustration that many patients attached little value to the treatment modality.

*‘Most of the time, this requires counselling. There are some who do not require or need drugs; they only need counselling. That is talking therapy. That way we know the extent of how they are affected. For such ones we schedule visits for them. We like to have a discussion with them.’*

**(48 years old CHEW)**

PHCW reported that a key aspect of treatment for depression in older people was to involve their family members including their children in the intervention process. This was often initiated by the PHCW making direct telephone contact with patients’ family and inviting them to join clinical meetings with the older person. Health workers reported that during these meetings, they would educate family members as to the medical nature of patients’ illness, as well as the important social determinants of patients’ illness. PHCWs suggested that these sessions also provided patients with the opportunity to express their personal needs for support directly to their family members. In addition, PHCWs reported providing information about what other tangible support family members could provide to help the older person with depression. Family sessions were seen as providing an opportunity for a reactivation of regular contacts between patients and their family members.

*‘On knowing that (he is depressed), I invited one of his children. I also invited the man (patient) and counselled him and let him know that what has happened has happened and that he should put his mind at rest. His daughter whom I invited was also counselled, that she shouldn't leave her father alone, that you should be there to take care of him and to make sure that he takes whatever drug was given to him.’*

**(56 years old CHO)**

Primary healthcare workers described how, as an adjunct to counselling and family sessions, prescription of mild analgesics as well as haematinics was provided to older people with depression. In addition, health workers reported occasional prescriptions of sedatives when patients’ depression symptoms included sleep problems. Participants reported rarely using antidepressants for the treatment of depression. As described by PHCW, the use of medications as an option for treating depression was in keeping with a wider existing practice of prescribing medications in the treatment of most ailments presenting in PHC.

*‘But for an elderly patient with depression, like the woman I just mentioned, I did not use anything for her. I only asked her to used paracetamol for headaches and I told her to get diazepam to correct her sleeping patterns. That was what I used for her until she got okay.’*

(**38 years old CHO**)

PHCWs reported that older people with depression often came to PHC with an expectation to receive prescription for drugs and/ or injection as part of treatment. They expressed concerns that older patients often made direct requests for prescription drugs even in situations where the health worker may have estimated that patients’ depression do not require medications. Participants also perceived that older people often do not expect to recover from their depression if treatment did not include medications or injections.

*‘Some of the challenges include them expecting you to give them medications. Some think psychoeducation is just an ordinary discussion. They believe everything wrong with them can only be sorted out with medications, whereas psychoeducation works better than drugs most times if they can adhere to it.’*

(**Interview, 49 years old CHO**)

### Treatment engagement

PHCWs reported that from their experience, older people with depression were relatively more inclined to participate actively in their treatment plans and remain engaged in treatment compared with younger patients. Patient factors identified by PHCW as facilitating of treatment engagement in PHC include the view that many older people perceived encounters with their PHCWs as opportunities for social engagement.

*‘…of course, they comply with treatment because most of them are less busy now, they don’t have so much doing, they come, in fact, they find it interesting. They have somebody to go and talk to, In fact, most of them, staying at home, they feel lonely. So, they believe when they come to hospital, they have someone to rub minds with.’*

(**38 years old CHO**)

### Theme three: Community outreach implications

This theme is related to PHCW experience and views about providing care and support for older people with depression outside the PHC and in patients homes and include their thoughts about the value of mobile technology for community-based care.

### Views on home-based care for older people

PHCWs workers reported that medical outreach to older people living in the community, including those with depression, was an expected component of their work role. However, many PHCW reported that their current community practice experience was limited to infectious diseases immunization work.

*‘We have done outreach about measles and other immunizations, and also (Mosquito) nets distribution’*.

(**31 years old CHEW**)

Some PHCW recalled positive previous experience of conducting community outreach for older people, including those living with depression. Health workers identified many advantages of this type of work, including overcoming barriers to accessing PHCs due to poor mobility. Also, PHCW reported that community outreach provided an opportunity to assess patients home environment, as well as immediate social support network. In addition, home visits to older people were perceived by PHCW as enhancing of therapeutic relationship as well as ensuring continuity of care.

*‘But because of their age and mobility, they may not be able to leave the house to seek medical care at the clinics. But if they are visited at home by medical personnel, they would be elated such that they will tell everyone who cares to listen that they were visited by health care professionals. They will be very happy and count themselves as part of the community.’*

(**48 years old CHO**).

Despite many perceived advantages of community outreach for older people with depression, healthcare workers contended that it was difficult to implement home visits for older people in the current context of primary care practice. Participants reported that this was due to lack of incentives, including provision of transportation facilities, to carry out home visits.

*‘What I can say that is hindering us to take care of them, is the transportation means to get to their homes, even the communication gap (means of communication). So if we have those things, it will make it easier for us to take care of them*.’

**(41 years old CHEW)**

Some PHCW reported previous experience with use of a mobile phone application that served as a clinical guide to mental health assessment and treatment in their primary care practice. They suggested that this application could be amended to include assessment and treatment for older peoples’ depression. The health workers suggested that a clinical decision-making application for older people with depression may include information about how patients and their family caregivers could conduct basic physical health checks at home and report results back to the healthcare worker for a comprehensive assessment and treatment decision.

*‘Maybe phones should be provided for the health workers and an application that would guide them in the treatment and care of the elderly patient be installed on the phones’*

**(51 years old CHO)**

## Discussion

PHCWs in the present study perceived that an often-late presentation of depression with a combination of mood, behavioural and cognitive symptoms made clinical assessment of older people with the condition a challenging activity. The common treatment practices identified were basic counselling and involving family in care. A perceived need to frequently prescribe sedatives, mild analgesics and vitamins because of patients’ expectation, as well as non-existence of community-based outreach programmes were identified as existing barriers to providing evidenced based continued care for older people with depression in the study settings. These barriers were thought to be surmountable by future deployment of smartphone applications with capability for remote diagnostic assessment and treatment.

Our findings are consisted with the qualitative research literature suggesting that primary care providers demonstrate negative attitudes to older people who present with mental health conditions(15–17). Notably, the literature is strikingly sparce on the experience of lay PHCW who cared for an older person with depression. In a systematic review of thirteen qualitative studies conducted across Western Europe, North America and Australia(15), the mostly specialist family physicians (GPs) perceived encounters with older people who presented with depression as time consuming, exasperating and emotionally draining. In a recent qualitative interview of 14 non-specialist public primary care doctors in Malaysia(16), assessment of depression in older people was perceived as time consuming and often involved several complex conversations with patients and their families. Overall, clinicians have often expressed worry that the amount of time required for clinical consultation with older people may set them behind their busy clinic schedule(17).

Similar to the present study, primary care providers in the studies reviewed Schuman and colleagues (2012) as well as others in the literature(18) considered a perceived vague or otherwise clinically unclear presentation of depression, as well as patients’ confrontational behaviour and resistance to clinicians’ perspectives about the mental health nature or treatment of their conditions as the most important barriers to working with older people who present with depression(15). A key difference is that our study sought to examine the experience and perception of lay PHCW who in Nigeria comprised CHOs and CHEWs. Participants in the present study perceived patients’ behaviour as difficult, and that they often held discordant views.

Providers in the present study also held the view that even when they felt confident in asking questions about important depression symptoms including especially suicide (which though may sometimes be considered a taboo topic in Nigeria), patients demonstrated resistance or provided ambiguous answers. It was difficult to identify other African studies of primary care providers’ perspectives of older peoples’ depression. One previous qualitative study of older peoples’ personal experience of depression(19) suggested that the taboo attached to the experience of suicidal thoughts and behaviour ensured that participants in rural Tanzania described these experiences in indirect terms, such as for example having ‘a wish to die’.

Similar to findings in rural Tanzania(19), providers in the present study reported frequent encounters with patients who presented with mostly bodily symptoms and those who were perceived to have poor memory and other cognitive dysfuntions. PHCW perceived that patients’ poor cognitive performance further impinged on providers ability to make a confident diagnostic assessment of depression. They particularly expressed difficulty in differentiating depression from dementia in patients presenting with more cognitive symptoms.

In all, PHCW in in the present study expressed greater confidence in their ability to help patients engage in treatment for depression than in assessment and diagnosis of the condition. They identified self-disclosure and narratives of personal experiences as tools to foster connections with patients. Therapy sessions were reported to be typically discussive and were perceived as effective in treating depression over several sessions. Previous qualitative descriptive studies as well as ethnographic investigations of experiences and perception of nurses who provided geriatric care to patients with depression(18, 20) found that nurses’ use of friendliness and light informal conversation helped in fostering social connections during clinical encounters by reducing professional barriers and enabling patients to become more involved in discussing personal issues related to their experience of depression. Over several treatment sessions, this approach was important in helping to build patient-provider therapeutic relationship(18). Perhaps because of this relationship, and as described by PHCW in the present study, older people with depression were perceived to be more engaged in their treatment than other patient populations seen in Nigeria primary care settings.

We identified existing barriers to providing evidenced based treatment for older people with depression in Nigerian primary care settings. For example, we found that it was part of patients’ expectation for staff to prescribe sedatives, mild analgesics, or vitamins even when PHCW has determined that psychosocial intervention was sufficient treatment for depression. Also, factors external to the control of the older person, such as distance to the PHC, mobility issues and reliance on availability of caregivers often led to disengagement from continued care as may be required for depression. Furthermore, even though desired by PHCW, there were no community-based outreach programmes to ensure continuity of care.

Globally, continuity of care is pivotal to the provision of effective mental health care for older people. In a previous chart review study at an outpatient mental health service for older people in Nigeria, we found that only 18.4% of registered patients attended scheduled follow-up care over an approximately 2-year period(21). Over half had dropped out of follow-up after approximately 1-3 irregular contacts with the service(21). In that study(21), financial difficulties, distant location of the service and long waiting times were the main reasons given by patient participants for dropping out of outpatient services for older people in Nigeria. In the circumstances of high rates of dropout from primary care services and absence of community-based outreach programmes to ensure continuity of mental health care, PHCW in the present study identified the introduction of smartphone-based applications with assessment and treatment capabilities as a way to overcome the barriers to continuity of evidence-based care for older Nigerians with depression and other mental health conditions.

This study, to the best of our knowledge, is the first qualitative study done to explore the perception and views of lay PHCW about management of older people presenting with depression in primary healthcare settings in Nigeria and sub-Saharan Africa. The main limitation of the present study is that it was conducted among staff in 10 PHCs in Ibadan Southwestern Nigeria, and as such findings may not be generalisable to all Nigerian primary health care settings.

## Conclusion

We have found that non physician PHCW perceived that patients’ poor cognitive performance which overlapped with those encountered in people with dementia, expectations to prescribe sedatives, analgesics and vitamins, as well as non-existence of community-based services were existing barriers to providing evidenced based continued care for older people with depression in Nigeria. Despite these barriers, PHCW in the study settings demonstrate awareness and willingness to provide treatment for older people with depression. It would appear from our findings that overcoming existing barriers may be key to expanding evidence based mental health care for older Nigerians with depression and other mental health conditions.

## Supplementary Material

Appendix

## Figures and Tables

**Figure 1:**
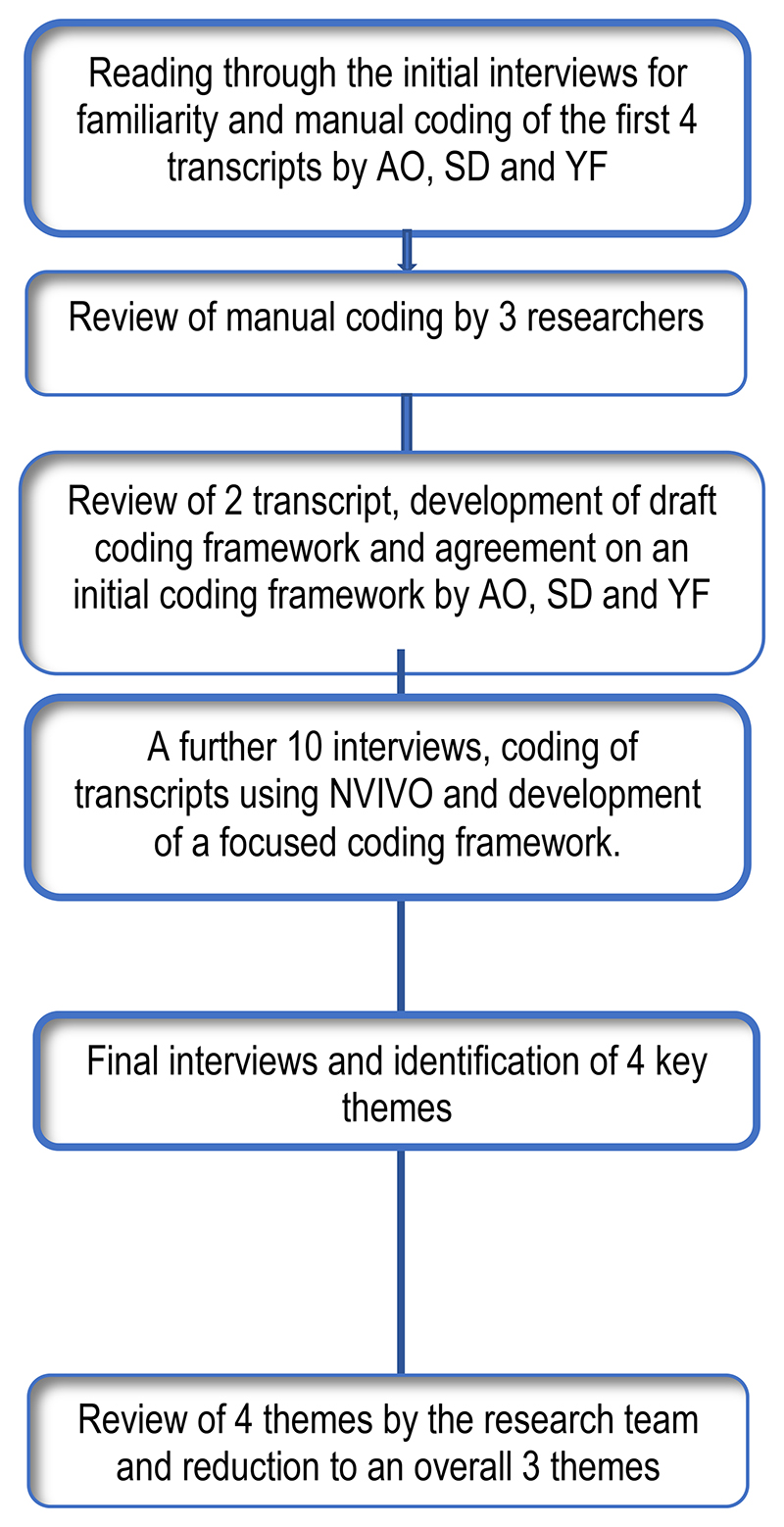
Process of qualitative data analyses

**Table 1:** Profile of primary healthcare clinics included in the study

Clinics	Patient flow per month	Rural/urban distribution
Aba Emu	1490	Rural
Alakia	1298	Rural
Ojoo	1200	Rural
Odo-Ona Elewe	623	Rural
Oranyan	520	Urban
Apete	500	Urban
Alegongo	500	Rural
Oniyanrin	500	Urban
Oke Adu	350	Urban
Sango	350	Urban

**Table 2:** Profile of primary health care workers selected for qualitative interviews and focus group.

CHARACTERISTICS	AVERAGE	RANGE
*Age*	44 years	31-56 years
*Years of work experience*	19 years	6-30 years
		
	**NUMBER**	**PERCENT**
*Sex*		
Female	18	75
Male	6	25
		
*Designation*		
Community health officers	14	58
Community health extension workers	10	42
*Location of practice*		
Rural	13	54
Urban	11	46

**Table 3:** Summary of themes from the experience of Community Health Officers (CHO) and Community Health Extension Workers (CHEW) who provided care for older people with depression in primary care settings.

THEMES	SOME ILLUSTRATIVE QUOTES
**Views about Depression** Characterised by mood, behavioural and cognitive symptoms which made clinical assessment difficult. Symptoms and clinical presentation overlapped with those seen in people with dementia. Bodily symptoms as a gateway to accessing primary health care clinics Personal economic challenges and lack of social support as key triggers of depression.	*‘You know, someone who presented with physiological health problems won’t give a lot of trouble compared to those with depression or other mental health problems. People with mental health challenges pose a lot of trouble. Sometimes, they could be irritable, angry, thinking someone is being rude to them.’* (**Interview 49 years old CHO**) *‘In a depressive (depressed) elderly, they usually have low mood, mood swings and withdraw (al) from daily activities. So, the one with depression is withdrawn and experiences low mood…, none depressed person don’t show these symptoms.’* (**Focus group 39 years old CHEW)** *‘I actually realize that most of the elderly, they have issues of their belief system. No matter what you try to tell them, they have a concept of what is wrong with them. They have this mindset of what is wrong with them which they want to tell you and they want u to believe it too. So, you need to find a way of trying to convince them to change their orientation about that thing that they believe. Sometimes they believe that it’s spiritual. Yes, “the awon aye” (A curse or bewitchment)..maybe from their families.* (**Interview 38 years old CHO**) *‘Hope you are not thinking of commuting suicide? that is the question they react to most of the time. It is always irritating to them, most of them will be like God forbid.’* (**Interview 37 years old CHEW**) *‘…she doesn’t talk well, if asked a question, she derails completely from the answer.’* (**Interview 38 years old CHO**) *‘Sometimes, you (Heath worker) may tell them something and they won’t hear because they were absent minded…Then, if you (Health worker) remind them that you (Health worker) initially asked if they have headache or insomnia, they’ll deny hearing the question….then you’ll know that the individual is challenged.’* (**Interview 49 years old CHO**) *‘There is also the peculiarity of dementia among them. So, they need someone to remind them if they have taken their drugs, including the clinicians.’* **(Interview, 56 years old CHO)** *‘When someone gets old, they forget things. This is called senile dementia. But if someone does not have the knowledge of depression, he might mistake or misdiagnose it as depression.’* **(Interview 51 years old CHO)** *‘He wasn’t violent, but he withdrew and was not eating. He has been on this for more than 2 months. But they didn’t know until he came to the clinic presenting a physical problem that it was detected that he had depression.’* (**Interview, 56 years old CHO**) *‘They (Patients) might say they have arthritis or body pain. If one isn’t thorough, he might not know that the patient is depressed.’* (**Interview 46 years old CHEW**) *‘The difference I know is that when you ask them about their children, they would tell you that they are alone. That would tell us that she has depression in the sense that there is no one to care for her and if she sees her children it would give her joy.’* (**Interview 41 years old CHEW**) *‘There was a woman who suffered economic reversals in her business. She could no longer care for her children, so this made her depressed.’* (**Interview, 48 years old CHO**)
**Treatment of depression in primary care** Counselling in the form of basic psychoeducation and advice Adjunctive use of sedatives and vitamins Involving patients’ family in their care Positive perception of treatment engagement	*‘Most of the time, this requires counselling. There are some who do not require or need drugs; they only need counselling. That is talking therapy. That way we know the extent of how they are affected. For such ones we schedule visits for them. We like to have a discussion with them.’* **(Interview, 48 years old CHEW)** *‘The first thing is their belief system. Sometimes they believe that it’s spiritual. Yes, ‘the awon aye’ (Spirit agents) trying to get them, maybe from their families. The only challenge for me is trying to bring them out of that belief system to the reality of what is happening to them.’* **(Interview, 38 years old CHO)** *‘She then told me that since the other children are not taking care of her, she thinks death is the best option. I told her death is not an option, and that d other children would take care of her, if they’re being told that it is their responsibility. I later called on the children that accompanied her, then told them what mama said n they promised to take care of her. They also asked her what she wanted. She told them what her late child does for her and they promised to do it. Mama told them she’ll be fine if they fulfil their promise.’* **(Interview, 49 years old CHO)** *‘On knowing that, I invited one of the children. I also invited the man and counseled him and let him know that what has happened has happened and that he should put his mind at rest. His daughter whom I invited was also counselled, that she shouldn’t leave her father alone, that you should be there to take care of him and to make sure that he takes whatever drug was given to him.’* **(Interview, 56 years old CHO)** *‘But for an elderly patient with depression, like the woman I just mentioned, I did not use anything for her. I only asked her to used paracetamol for headaches and I told her to get diazepam to correct her sleeping patterns. That was what I used for her until she got okay.’* (**Interview, 38 years old CHO**) *‘I gave her some homework and amitriptyline because of lack of sleep.’* (**Interview, 37 years old CHEW**) *Some of the challenges include them expecting you to give them medications. Some think psychoeducation is just an ordinary discussion. They believe everything wrong with them can only be sorted out with medications, whereas psychoeducation works better than drugs most times if they can adhere to it.’* (**Interview, 49 years old CHO**) *‘So, I realize that she’s having some symptoms of depression, and I tried to book her for counselling sessions. She said she’s having pain instead and that I needed to give her some mild analgesics to at least be fine.’*(**Interview, 38 years old CHO**) *‘We would tell them that due to their age there is need for them to be coming for checkup, and of course, they comply, because most of them are less busy now, they don’t have so much doing, they come, in fact, they find it interesting. They have somebody to go and talk to, In fact, most of them, staying at home, they feel lonely. So, they believe when they come to hospital, they have someone to rub minds with.’* (**Interview, 38 years old CHO**) *‘Though they are cooperative, there are times when the children might take them away to another place. Like the example I cited earlier, the woman used to live alone. But when the children saw that she was getting better, they took her to another state (County)’* (**Interview, 46 years old CHEW**).
**Community outreach** Expected practice, but currently limited to infectious diseases immunization. No incentives to implement for older peoples’ mental health conditions in current practice. Mobile technology may circumvent current barriers to implementation in future practice.	*‘Normally, going for home visit is part of our work, it is inside the standing order that we have with us.’* (**Interview, 41 years old CHO**) *‘We have done outreach about measles and other immunizations, and also (Mosquito) nets distribution’.* (**Focus group, 31 years old CHEW**) *‘But because of their age and mobility, they may not be able to leave the house is to seek medical care at the clinics. But if they are visited at home by medical personnel, they would be elated such that they will tell everyone who cares to listen that they were visited by health care professionals. They will be very happy and count themselves as part of the community.’* (**Interview, 48 years old CHO**). *‘What I can say that is hindering us to take care of them, is the transportation means to get to their homes, even the communication gap (means of communication). So if we have those things, it will make it easier for us to take care of them.*’ **(Interview, 41 years old CHEW)** *I feel that if going to visit will be difficult, calling would serve as a solution to that to some extent. Though, it is not a full solution; we will still need to visit.* **(Interview, 56 years old CHO)** *I treated an elderly patient under remote consultation. When we have some with chronic diseases and they would like to be treated on the mobile (over the phone, I guess), we accept such.* **(Interview, 51 years old CHO)** *‘Maybe phones should be provided for the health workers and an application that would guide them in the treatment and care of the elderly patient be installed on the phones’* **(Interview, 51 years old CHO)** *‘If it’s possible for the app to have something like a voice note, where u can play to them, in a language like in Yoruba. If it’s possible the app is in a way that if it can speak out to them. Any instruction you want to give them, maybe app first reads it out and do a level of reinforcement on it. This might be attractive to the elderly.’* **(Interview, 51 years old CHO)**

## Keypoints

- Most mental healthcare in Nigeria is sought in primary healthcare.
- Primary healthcare workers (PHCW) hold negative views about older people.
- Late-life depression overlaps with dementia and is a barrier to accurate diagnosis.
- Though authorised, PHCW rarely prescribed antidepressants for depression.
