# Supplementary material for: The care of older people with depression in Nigeria: qualitative exploration of the experience of lay providers in primary care settings
Source: Int J Geriatr Psychiatry. Author manuscript; Available in PMC 2024 Sep 15. (PMC7616446; doi:10.1002/gps.6147)
Supplement: Appendix [file EMS198573-supplement-Appendix.docx]

**Appendix 1: Draft interview guidelines for CHEWs and CHO’s who recently identified and treated an older person with depression.**

| **S.N** | **THEMES AND POSSIBLE PROMPT** | **FURTHER PROBES** |
| --- | --- | --- |
| 1. | **General** |  |
| 1.1 | *Interview:*  Can you tell me a little bit about your normal work duties? | |
| 1.2 | Can you tell me a little about your experience with assessing and treating/providing support for elderly people? | What are the main differences if you are assessing and treating an elderly person with a mental health condition compared with non-mental health condition? |
| 1.3 | *Interview:*  You recently assessed and treated (Mr/Mrs….) an older/elderly person with depression in your clinic –  Can you tell me a what you did in your work with him/her? | In your experience, what is different about depression in older people compared to younger people?  -Explore examples of differences |
| 2. | **Barriers to care of an older person with depression** |  |
| 2.1. | *Interview:*  What difficulties did you experience in identifying and treating depression in an older/elderly person? | How easy or difficult was it for you to ask your older/elderly patients about their symptoms of depression?  Probe for examples of questions patients consider as sensitive. |
| 2.2. | *Interview:*  Thinking about your work duties and the way it is organised, is there anything which affects your being able to support (i.e., assess and treat) older/elderly people with depression? | Probe in areas of:   - Work load, staff strength general clinic structure - Availability of space for private consultation with patients.   Are there other factors (may or may not be related to your clinic setting) that you would see as hindering your being able to identify and treat an older/elderly person with depression? |
| 3. | **Facilitators of care of an older person with depression** |  |
| 3.1. | *Interview:*  What factors would you see as helping your being able to identify and treat an older/elderly person with depression in your clinic? | What other factors (may or may not be related to your clinic setting) would you see as helping your being able to identify and treat an older/elderly person with depression? |
| 4. | **Perception about home-based care for older people** |  |
| 4.1. | *Interview:*  We are planning to develop a model that would allow you to provide the treatment and support needed by an older/elderly person with depression in their own home. This might involve use of the mhGAP on smartphone or tablet.  What are your thoughts about this idea? | How do we make the idea work? |
| 4.2. | What needs to change to make it work? | (If not answered by response, explore –  What would be helpful to include in a home-based treatment for an older/elderly person with depression?’  [views on features and why they are likely to be helpful?]  [explore more about examples]  Think about any other areas of older/elderly peoples’ day to day life, what else would be important to include in your ideal home-based care? |

**Thank you for your time.**

**Appendix 2: Draft Focus guidelines for CHEWs and CHO’s who recently identified and treated an older person with depression.**

| **S.N** | **THEMES AND POSSIBLE PROMPT** | **FURTHER PROBES** |
| --- | --- | --- |
| 1. | **General** |  |
| 1.1 | *Focus group:*  What are the main differences if you are assessing and treating an elderly person with a mental health condition compared with non-mental health condition? | -Explore examples of differences |
| 1.2 | *Focus group:*  In your experience, what is different about mental health condition/depression in older people compared to younger people? | -Explore examples of differences |
| 2. | **Barriers to care of an older person with depression** |  |
| 2.1. | *Focus group:*  Thinking about your work duties and the way it is organised, is there anything which affects your being able to support (i.e., assess and treat) older/elderly people with depression? | Probe in areas of:   - Work load, staff strength general clinic structure - Availability of space for private consultation with patients.   Are there other factors (may or may not be related to your clinic setting) that you would see as hindering your being able to identify and treat an older/elderly person with depression? |
| 3. | **Facilitators of care of an older person with depression** |  |
| 3.1. | *Focus group:*  What factors would you see as helping your being able to identify and treat an older/elderly person with depression in your clinic? | What other factors (may or may not be related to your clinic setting) would you see as helping your being able to identify and treat an older/elderly person with depression? |
| 4. | **Perception about home-based care for older people** |  |
| 4.1. | *Focus groups:*  We are planning to develop a model that would allow you to provide the treatment and support needed by an older/elderly person with depression in their own home. This might involve use of the mhGAP on smartphone or tablet.  What are your thoughts about this idea? | How do we make the idea work? |
|  | What needs to change to make it work? | What would be helpful to include in a home-based treatment for an older/elderly person with depression?’  [views on features and why they are likely to be helpful?]  [explore more about examples]  Think about any other areas of older/elderly peoples’ day to day life, what else would be important to include in your ideal home-based care? |

**Thank you for your time.**
